# Marek’s Disease Virus (MDV) Meq Oncoprotein Plays Distinct Roles in Tumor Incidence, Distribution, and Size

**DOI:** 10.3390/v17020259

**Published:** 2025-02-14

**Authors:** Dharani K. Ajithdoss, Yifei Liao, Sanjay M. Reddy, Blanca Lupiani

**Affiliations:** Department of Veterinary Pathobiology, College of Veterinary Medicine & Biomedical Sciences, Texas A&M University, College Station, TX 77843, USA; dajithdoss@gmail.com (D.K.A.); yliao8@bwh.harvard.edu (Y.L.)

**Keywords:** Marek’s disease virus, MDV, Meq, DNA-binding domain, transcriptional regulatory domain, transformation, pathogenesis

## Abstract

Marek’s disease (MD), characterized by the rapid onset of T-cell lymphomas in chickens, is caused by *Mardivirus gallidalpha2*, an oncogenic alphaherpesvirus commonly known as Marek’s disease virus (MDV). MDV encodes a bZIP protein, Meq, which contains a bZIP domain (basic DNA-binding and leucine zipper dimerization domain) at the amino terminus and a transcriptional regulatory domain at the carboxyl end. Meq can transform murine and chicken fibroblasts in vitro and is essential for tumor formation in chickens. Meq homodimerization and heterodimerization through its bZIP domain are involved in Meq-mediated transformation. However, the role of Meq DNA-binding and transcriptional regulatory domains in transformation has not been investigated. In this study, we constructed recombinant Md5 (very virulent MDV) viruses expressing chimeric Meq proteins generated by swapping the DNA-binding and transcriptional regulatory domains of Meq of Md5 and vaccine (CVI988/Rispens) strains. Our results show that these recombinant viruses, rMd5-Md5/CVI-Meq (Md5 DNA-binding domain and CVI transcriptional regulatory domain) and rMd5-CVI/Md5-Meq (CVI DNA-binding domain and Md5 transcriptional regulatory domain), replicated at levels similar to parental rMd5 in cell culture and chickens and could transmit efficiently among chickens. Interestingly, parental rMd5 and chimeric viruses exhibited distinct pathogenic phenotypes in chickens: rMd5 caused 100% mortality, a moderate level of tumor incidence in visceral organs and small visceral tumors; rMd5-Md5/CVI-Meq caused 100% mortality, a high level of tumor incidence in visceral organs, and very large visceral tumors; while rMd5-CVI/Md5-Meq caused an average of 37% mortality, rarely induced tumors in visceral organs, and the visceral tumors were small. In conclusion, our study suggests that the DNA-binding domain of Meq plays an essential role in transformation (tumor incidence), while the transcriptional regulatory domain of Meq influences the distribution and size of MDV-induced tumors.

## 1. Introduction

*Mardivirus gallidalpha2* [1], also known as Marek’s disease virus (MDV), belongs to the genus *Mardivirus* in the family *Orthoherpesviridae*. MDV infection causes a lymphoproliferative disease resulting in paralysis, immunosuppression, blindness, and significant mortality in unvaccinated chickens [2]. MDV strains vary greatly in their virulence and are classified into pathotypes: mild (m), virulent (v), very virulent (vv), and very virulent plus (vv+), depending on the degree of disease caused in vaccinated chickens [3]. Currently, several vaccines exist that protect chickens against the development of lymphomas, but they do not provide sterilizing immunity; thus, field strains replicate in vaccinated chickens, leading to the evolution of MDV toward greater virulence [3]. Insights into the mechanisms of transformation by MDV will provide information that could be exploited in designing better vaccines. 

The MDV genome consists of a unique long (U_L_) and a unique short (U_S_) region, both flanked by inverted repeat regions named terminal repeat long (TR_L_), internal repeat long (IR_L_), terminal repeat short (TR_S_), and internal repeat short (IR_S_), respectively [4]. Two copies of *meq*, the gene that encodes MDV oncoprotein Meq, are located in the **M**DV ***E****co***Q** fragments within the long repeat regions (TR_L_ and IR_L_). This oncogene was first identified based on the observation that *Eco*Q transcripts were abundantly present in MDV-transformed T-cell lines and tumors [5]. Meq is a 339 amino acid long bZIP (basic leucine zipper) protein, characterized by a bZIP DNA-binding and dimerization domain (amino acids 1–120) at its amino (N) terminus, and a transcriptional regulatory domain (amino acids 121–339), at its carboxy (C) end [5,6]. The structure and function of Meq closely resemble c-Jun, a member of the family of activator protein 1 (AP-1) transcription factors [5]. In addition, the bZIP and transcriptional regulatory domains of Meq and c-Jun can functionally complement each other in transformation [7]. Like c-Jun, Meq forms homodimers with itself and heterodimers with other bZIP proteins to suppress and activate transcription, respectively [8,9,10]. Meq/c-Jun heterodimers preferentially bind cyclic AMP (CRE)- and 12-O-tetradecanoylphorbol 13-acetate responsive element (TRE)-like sequences, called MERE (**Me**q **r**esponsive **e**lement) I, while Meq/Meq homodimers preferentially bind a consensus sequence ACACACA, called MERE II [8,9,10]. Meq localizes predominantly to the nucleoplasm and nucleolus during all cell cycle phases. Two basic signal sequences, BR1 (30-RRKKRK-35) and BR2 (62-RRRKRNRDAARRRRRKQ-77), present at the N-terminus of Meq, are required for its localization [11]. While BR1 and BR2 function as nuclear localization signals (NLS), BR2 also functions as Meq’s sole nucleolar localization signal (NoLS) [11]. Meq has also been characterized as a substrate of cellular protein kinases (including CDK2, PKA, PKC, and MAPK) and MDV-encoded U_S_3 protein kinase [12,13]; specifically, CDK2 was shown to phosphorylate Meq at serine residue 42, resulting in the translocation of Meq to the cytoplasm [12]. We have recently shown that the bZIP domain of Meq is important for its interaction with chicken histone deacetylase 1 and 2 (chHDAC1 and 2) [14] and is also involved in Meq-mediated proteasomal degradation of chHDAC1 and 2, as well as other ubiquitinated host proteins [14]. On the other hand, the proline repeat-rich C-terminus of Meq has transcriptional regulatory properties, and the last 33 amino acids (307–339), though not sufficient, are critical for its transactivation function [6]. 

Overwhelming evidence shows that Meq plays an important role in MDV oncogenesis. Meq was shown to be required to maintain the transformation status of MSB-1 cells, an MDV lymphoblastoid cell line, since the induction of RNA antisense to *meq* inhibited the proliferation of MSB-1 and reduced colony formation in soft agar assay [15]. When overexpressed, Meq transforms rat fibroblasts (Rat-2 cells), mouse fibroblasts (NIH3T3 cells), and immortalized chicken fibroblasts (DF-1 cells) [16,17,18]. In addition, Meq protects Rat-2 cells from apoptosis induced by tumor necrosis factor-alpha (TNF-α), C2-ceramide, serum starvation, and UV irradiation [16]. Our previous study, using a recombinant vv MDV *meq* null virus (rMd5ΔMeq), provided compelling evidence that Meq is essential for transforming T lymphocytes in chickens [19]. Additional evidence that supports Meq involvement in transformation came from a study with a Meq mutant virus, which was unable to interact with C-terminal-binding protein (CtBP) and was found to be non-oncogenic in chickens [20]. It has also been suggested that Meq transforms via the v-Jun pathway [17], and we and others have demonstrated that both homo- and heterodimerization of Meq contribute to the transformation of lymphocytes in chickens [21,22,23]. 

Sequence analysis of *meq* genes has shown that amino acid changes in Meq correlate with the virulence of MDV strains [24]. We have previously shown that Meq from the non-oncogenic vaccine strain CVI988/Rispens is generally a weak transactivator, although it retains the ability to transform fibroblasts [18]. We also showed that a recombinant vvMDV (Md5 strain) expressing the Meq protein from vaccine strain CVI988 (CVI-Meq) showed significantly reduced pathogenesis [25]. However, in the background of RB1B, another vvMDV, CVI-Meq rendered the virus apathogenic [26].

It remains unknown whether the amino acid differences in the DNA-binding or transcriptional regulatory domains of Meq are critical for transformation. To address this, we constructed two recombinant Md5 viruses (rMd5-Md5/CVI-Meq and rMd5-CVI/Md5-Meq) with chimeric Meq proteins by swapping the bZIP and transcriptional regulatory domains between Md5-Meq and CVI-Meq and evaluated their pathogenicity in chickens. An additional recombinant virus, rMd5-CVI-Meq, in which Md5-Meq was replaced with CVI-Meq, was used as control. Our results show that all recombinant viruses grew at levels comparable to parental rMd5 in cell culture and chickens during early cytolytic infection and were transmitted horizontally. rMd5-Md5/CVI-Meq caused 100% Marek’s disease (MD), and tumors were found in peripheral nerves and various organs, such as the heart, spleen, kidneys, and gonads. Interestingly, these visceral tumors were unusually large when compared to tumors induced by parental rMd5. On the other hand, rMd5-CVI/Md5-Meq caused an average of 37% MD in chickens, and gross lesions were primarily in the peripheral nerves. Based on these findings, we propose that the Meq DNA-binding domain determines tumor incidence, while the Meq transcriptional regulatory domain regulates the distribution and size of tumors in chickens.

## 2. Results

**Figure 1:**
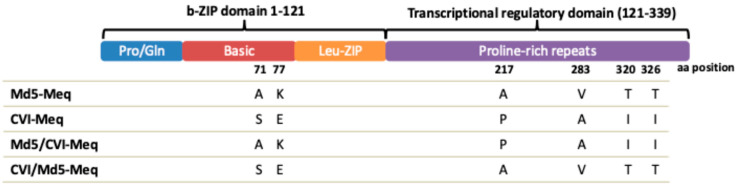
Schematic representation of Md5, CVI, and chimeric Meq proteins. Compared to Md5-Meq, CVI-Meq contains 2 amino acid differences in the basic DNA-binding domain and 4 amino acid differences in the transcriptional regulatory domain. Chimeric Meq proteins were constructed by swapping the basic DNA-binding domain and transcriptional regulatory domain of Md5-Meq and CVI-Meq. Numbers indicate the amino acid position.

**Figure 2:**
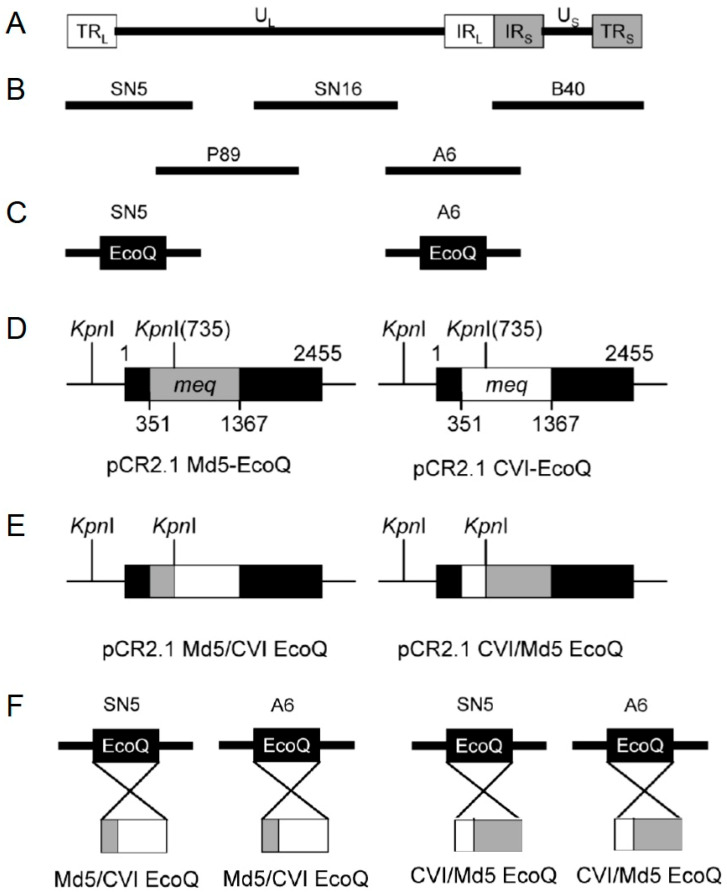
Construction of chimeric viruses using Md5 cosmid clones. (**A**) The MDV genome consists of a unique long (U_L_) and unique short (U_S_) region flanked by repeat regions (TR_L_, IR_L_, IR_S_, and TR_S_). (**B**) Five overlapping cosmid clones that cover the entire Md5 genome were used for the construction and recovery of chimeric viruses. (**C**) Cosmid clones SN5 and A6 contain the *Eco*Q fragment, in which *meq* is located. (**D**) Two *Kpn*I sites are presented in pCR2.1 Md5-EcoQ and pCR2.1 CVI-EcoQ vectors, one each in vector and *meq* sequence. (**E**) *Kpn*I fragments between pCR2.1 Md5-EcoQ and pCR2.1 CVI-EcoQ vectors were exchanged to generate pCR2.1 Md5/CVI-EcoQ and pCR2.1 CVI/Md5-EcoQ. (**F**) Parental Md5-EcoQ fragments in SN5 and A6 cosmid clones were replaced with Md5/CVI-EcoQ or CVI/Md5-EcoQ to generate chimeric cosmids.

**Construction of Meq chimeric viruses**. We have previously demonstrated that Meq plays a direct role in MDV oncogenesis [19]. Intriguingly, although CVI988/Rispens, an MDV vaccine strain, codes for Meq, it does not cause tumors. CVI-Meq contains two and four amino acid differences in the DNA-binding region and transcriptional regulatory domain, respectively, relative to Md5-Meq (Figure 1). To study the function of Meq DNA-binding and transcriptional regulatory domains in transformation, we constructed three recombinant Md5 viruses: rMd5-Md5/CVI-Meq (Meq DNA-binding domain of Md5 and Meq transcriptional regulatory domain of CVI), rMd5-CVI/Md5-Meq (Meq DNA-binding domain of CVI and Meq transcriptional regulatory domain of Md5) (Figure 2), and rMd5-CVI-Meq (Md5-Meq was replaced with CVI-Meq). To confirm that no unforeseen major DNA rearrangements occurred during the generation of viruses by homologous recombination of the five cosmid clones, the integrity of the viral genomes was confirmed by Southern blot analysis. DNA isolated from duck embryonic fibroblasts (DEF) infected with recombinant viruses was digested with *EcoR*I and transferred to nylon membranes, which were then probed with either radio-labeled viral genome (Figure S1A) or *Eco*Q fragment (Figure S1B). As shown in Figure S1, the genomes of rMd5 (lane 1), rMd5-CVI-Meq (lane 2), rMd5-Md5/CVI-Meq (lane 3), and rMd5-CVI/Md5-Meq (lane 4) showed identical *Eco*RI digestion patterns. Th expression of Meq in DEF infected with recombinant viruses was examined using rabbit anti-Meq polyclonal antibody and, as expected, was predominantly found in the nucleus. This demonstrates that CVI-Meq and chimeric Meq proteins (Md5/CVI-Meq and CVI/Md5-Meq) were expressed from recombinant viruses with no change in their cellular localization properties.

**Figure 3:**
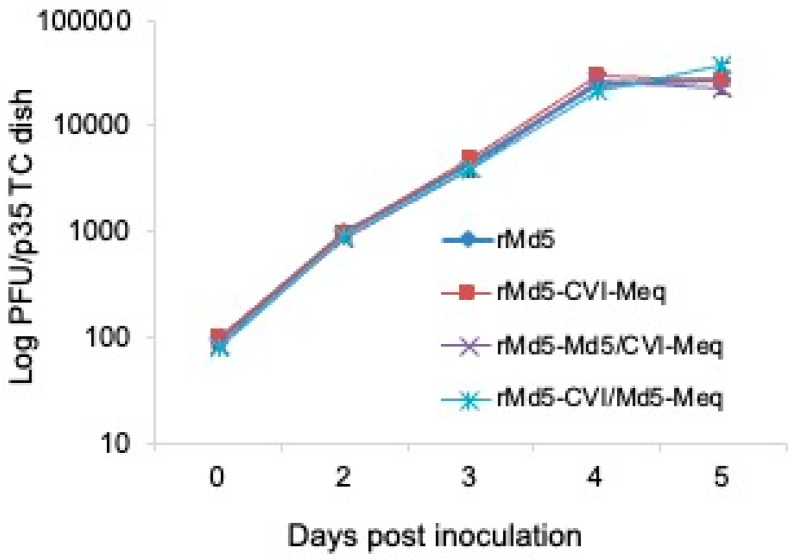
In vitro growth kinetics of parental and chimeric viruses. DEF were infected with 100 PFU of the indicated viruses. Cells were harvested on days 2, 3, 4, and 5 post-infection and virus titers were determined on fresh DEF. Data are presented as mean ± standard error of two independent experiments performed in duplicate.

**In vitro and in vivo growth properties of Meq recombinant viruses**. Meq is non-essential for MDV replication [19]. To investigate whether chimeric Md5/CVI-Meq or CVI/Md5-Meq had any effect on MDV in vitro replication, DEF in 35 mm dishes were infected with 100 plaque-forming units (PFU) of rMd5, rMd5-CVI-Meq, rMd5-Md5/CVI-Meq, and rMd5-CVI/Md5-Meq viruses. As expected, the in vitro growth properties of all chimeric viruses were comparable to those of parental rMd5 at all time points studied (days 2, 3, 4, and 5) (Figure 3), confirming that none of the Meq proteins examined affected viral replication in vitro.

To further investigate the role of chimeric Meq proteins in early cytolytic infection in chickens, two chickens from each group in experiment 1 were euthanized at 6 days post-inoculation (dpi), and lymphoid organs (spleen, thymus, and bursa) were collected and examined for virus replication by immunohistochemistry with a monoclonal antibody that targets pp38, an MDV early protein. All recombinant viruses replicated to similar levels, as no differences in pp38 antigen expression were observed in all three organs examined, suggesting that the early cytolytic replication of MDV in chickens was not affected by any of the Meq proteins examined. 

**Reactivation ability of Meq recombinant viruses.** To determine if any of the Meq proteins examined affect the ability of recombinant viruses to reactivate from peripheral blood lymphocytes (PBL), buffy coats were isolated at 35 dpi from three chickens per group in Experiment 1. 1 × 10^6^ PBL were co-cultivated with confluent monolayers of DEF seeded in 35 mm culture dishes, and plaques were counted 7 days later. As shown in Table 1, the number of plaques obtained from parental rMd5 and rMd5-Md5/CVI-Meq was significantly higher than from rMd5-CVI-Meq, and rMd5-CVI/Md5-Meq. Since the reactivation of the virus at 35 dpi (during the transformation stage) is proportional to the number of transformed cells in PBL, these results suggest that amino acid differences found in the DNA-binding domain of Meq play an important role in the transformation of lymphocytes. 

**Figure 4:**
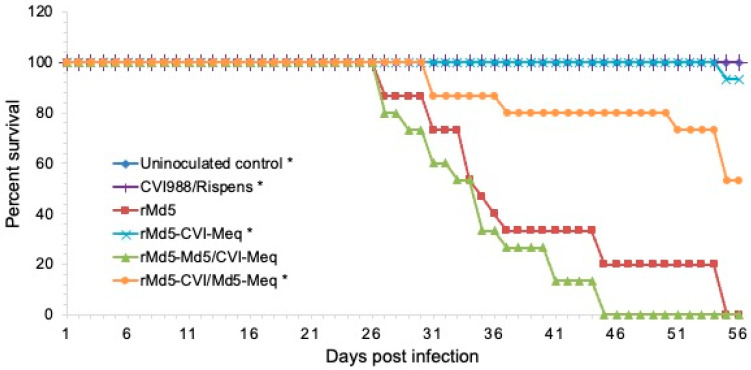
Survival curve of parental and chimeric viruses (Experiment 1). Specific pathogen-free chickens were inoculated with 5000 PFU of the indicated viruses and were maintained in isolation units for 8 weeks. The daily mortality pattern for each virus was recorded and the survival percentage for each group is shown on the *Y*-axis. Trends of chicken survival over time were examined with LogRank and Wilcoxon tests. A value of *p* < 0.05 was considered statistically significant when compared to the parental rMd5 virus. * = *p* < 0.001.

**Pathogenicity of Meq chimeric viruses.** To examine the ability of the various Meq proteins to induce tumors, chickens were inoculated with 5000 PFU of the respective recombinant viruses and monitored for MD mortality and tumors for 8 weeks (Experiment 1). Chickens in the uninoculated and vaccine (CVI988/Rispens) inoculated control groups did not die (Figure 4) or develop MD (Table 2). Chickens inoculated with rMd5-CVI-Meq and rMd5-CVI/Md5-Meq showed 7% and 47% mortality (Figure 4) and MD (Table 2), respectively. In stark contrast, 100% of chickens inoculated with rMd5-Md5/CVI-Meq and rMd5 died (Figure 4) and developed MD (Table 2) before the completion of the experiment.

**Figure 5:**
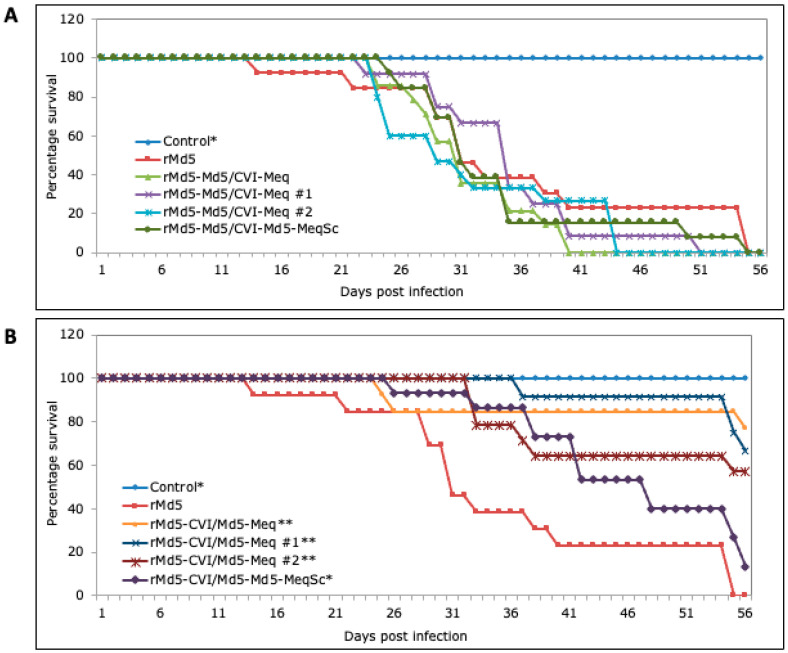
Survival curves of parental, chimeric, and single-copy revertant viruses (Experiment 2). Specific pathogen-free chickens were inoculated with 5000 PFU of the indicated viruses and were maintained in isolation units for 8 weeks. Control groups consisted of chickens inoculated with parental rMd5 virus, as well as uninoculated chickens. The daily mortality pattern for each virus was noted and the survival percentage for each virus is shown on the *Y*-axis. (**A**) Survival curves for uninoculated control group (control), rMd5 parental virus, and Md5/CVI-Meq recombinant viruses. (**B**) Survival curves for uninoculated control group (control), rMd5 parental virus, and CVI/Md5-Meq recombinant viruses. Trends of chicken survival over time were examined with LogRank and Wilcoxon tests. A value of *p* < 0.05 was considered statistically significant when compared to the parental rMd5 virus. * = *p* < 0.05 ** = *p* < 0.001.

To confirm that the differences in pathogenicity were due to chimeric Meq proteins and not to unforeseen mutations in other regions of the MDV genome, two additional rMd5-Md5/CVI-Meq and rMd5-CVI/Md5-Meq recombinant viruses (#1 and #2) were generated in two independent transfections, and their pathogenesis was examined in chickens (experiment 2). Inoculation of chickens with rMd5-Md5/CVI-Meq #1 and #2 resulted in 100% mortality (Figure 5A) and MD (Table 2), while mortality (Figure 5B) and MD incidence (Table 2) in chickens inoculated with rMd5-CVI/Md5-Meq #1 and #2 were 33 and 43%, respectively. In addition, two single-copy revertant viruses, rMd5-Md5/CVI-Md5-MeqSc and rMd5-CVI/Md5-Md5-MeqSc, in which one copy of Md5-*meq* was restored, were generated by co-transfection of viral DNA from rMd5-Md5/CVI-Meq or rMd5-CVI/Md5-Meq with the Md5-EcoQ fragment. When tested in chickens, single-copy revertant rMd5-Md5/CVI-Md5-MeqSc and rMd5-CVI/Md5-Md5-MeqSc viruses caused MD in 100% and 93% of chickens, respectively (Table 2).

**Figure 6:**
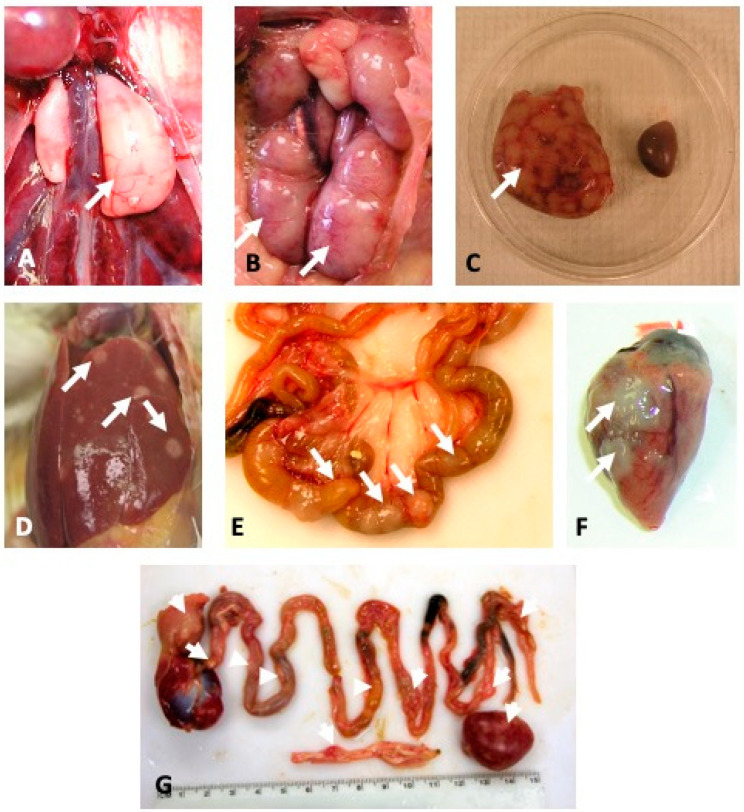
Visceral tumors in rMd5-Md5/CVI-Meq inoculated chickens. (**A**) Testicles; (**B**) ovary and kidney; (**C**) spleen with uninoculated control on the right; (**D**) liver; (**E**) tumors in the intestine; (**F**) heart; (**G**) gastrointestinal tract and spleen. Tumors are indicated by arrows. Visceral tumors caused by rMd5 and rMd5-CVI/Md5-Meq are too small to appreciate in a picture and are not shown.

Interestingly, the visceral tumor incidence, distribution, and size caused by rMd5-Md5/CVI-Meq were very different from rMd5 and rMd5-CVI/Md5-Meq (Table 3). In addition to gross lesions in peripheral nerves, rMd5-Md5/CVI-Meq induced high levels of lymphoid tumors in the heart (43%), spleen (39%), liver (9%), gonads (16%), and kidney (46%), and were of unusually large size (Figure 6). On the other hand, rMd5 primarily induced gross lesions in peripheral nerves and small visceral tumors in the heart (25%), spleen (14%), liver (4%), gonads (4%), and kidney (4%). rMd5-CVI/Md5-Meq induced tumors in the heart (2%) and spleen (2%) and did not cause tumors in the liver, gonads, and kidney. In addition, rMd5-CVI-Meq inoculated chickens did not develop any tumors in visceral organs, and gross lesions were restricted to peripheral nerves. Histological examination of affected nerves indicated that rMd5 and rMd5-Md5/CVI-Meq infected chickens had type A lesions, while chickens infected with rMd5-CVI/Md5-Meq had mostly type C lesions. Lesions in the positive chicken inoculated with rMd5-CVI-Meq were also type C.

These data collectively suggest: (1) the basic DNA-binding domain of Meq plays an important role in MDV transformation (tumor incidence); (2) the transcriptional regulatory domain of Meq influences the distribution and size of MDV-induced tumors; and (3) a combination of sequences in the Meq basic DNA-binding domain from Md5 and the transcriptional regulatory domain from CVI988/Rispens results in the development of large and widely distributed visceral tumors using rMd5 as the genetic background.

**Figure 7:**
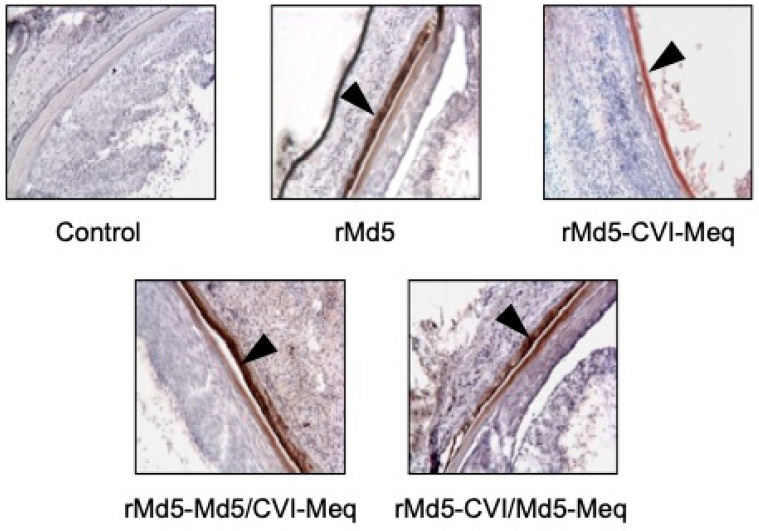
Viral replication in infected chickens’ feather follicular epithelium (FFE). The skin was collected from two randomly selected chickens in each group at the time of death or the end of the experiment, processed, and subjected to immunohistochemistry with mouse anti-pp38 monoclonal antibody to detect virus replication in FFE. Areas with virus replication are brown in color (black arrowhead). Representative images from each group are shown.

**Effect of chimeric Meq proteins on virus transmission in vivo.** Fully productive replication of MDV occurs only in feather follicular epithelium (FFE), where stable and fully infectious viruses are produced and released in dander, serving as a source of infection for other susceptible chickens. Therefore, we investigated whether rMd5-Md5/CVI-Meq and rMd5-CVI/Md5-Meq viruses replicate in FFE, an indication of transmissibility. To this end, the skin of two randomly selected chickens per group was examined for the presence of virus by IHC with mouse anti-pp38 monoclonal antibody. As shown in Figure 7, similar levels of positive pp38 staining were observed in the FFE of all viruses inoculated chickens. In addition, recombinant viruses were isolated from PBLs of contact chickens (uninoculated and co-housed with infected chickens). These results indicate that chimeric Meq proteins do not impact the transmission of Meq recombinant viruses among chickens.

## 3. Materials and Methods

**Cell culture**. Transfections to recover recombinant viruses were carried out using primary chicken embryonic fibroblasts (CEF) prepared from specific pathogen-free (SPF) chicken embryos. Generation of virus stocks, titration, growth kinetics, and reactivation assays were carried out using primary duck embryonic fibroblasts (DEF). CEF and DEF were cultured in Leibowitz–McCoy (LM) medium, supplemented with penicillin–streptomycin and 5% newborn calf, at 37 °C in the presence of 5% CO_2_. 

**Generation of cosmid clones and recovery of recombinant viruses**. MDV cosmid clones SN5, P89, SN16, A6, and B40 from the very virulent (vv) strain Md5 were used to generate recombinant Md5 viruses [27] (Figure 2A,B). The *Eco*Q fragment located in cosmid clones SN5 and A6 contains the *meq* gene (Figure 2C) and was replaced with Md5/CVI or CVI/Md5 *Eco*Q fragments using a previously described strategy [21,27,28]. Briefly, the Md5 *Eco*Q fragment was released from cosmid clones SN5 and A6 using the recA-assisted restriction endonuclease (RARE) method and was subsequently cloned into the pCR2.1 vector (Invitrogen, Carlsbad, CA) to generate pCR2.1 Md5-EcoQ. *Eco*RI-digested SN5 and A6 cosmid clones were re-ligated, generating SN5ΔEcoQ and A6ΔEcoQ. pCR2.1 CVI-EcoQ containing the *Eco*Q fragment from vaccine strain CVI988/Rispens was constructed following the same procedures. pCR2.1 Md5-EcoQ and pCR2.1CVI-EcoQ vectors were digested with *Kpn*I (Figure 2D) and the released fragments swapped to generate pCR2.1 Md5/CVI-EcoQ and pCR2.1 CVI/Md5-EcoQ (Figure 2E). Plasmids containing chimeric *Eco*Q fragments were then digested with *Eco*RI and the released fragment was cloned into A6ΔEcoQ and SN5ΔEcoQ to generate A6-Md5/CVI-Meq, SN5-Md5/CVI-Meq, A6-CVI/Md5-Meq, and SN5-CVI/Md5-Meq (Figure 2F).

Infectious rMd5-Md5/CVI-Meq and rMd5-CVI/Md5-Meq viruses were generated as previously described [21,27,28]. To generate rMd5-Md5/CVI-Meq, 500 ng of *Not*I-digested cosmids SN15, P89, B40, SN5-Md5/CVI-Meq, and A6-Md5/CVI-Meq were co-transfected, by the calcium phosphate method [29] into CEF seeded onto 60 mm dishes. Four days later, cells were trypsinized and seeded onto a 100 mm dish. Following the appearance of viral plaques, viral stocks were made using DEF. Additionally, to confirm that the observed phenotypes were not due to unintended alterations in the virus genome during the recombination process, two independent transfections for both chimeric viruses were carried out, and these were termed rMd5-Md5/CVI-Meq #1, rMd5-Md5/CVI-Meq #2. A similar procedure was followed to recover infectious rMd5-CVI/Md5-Meq, rMd5-CVI/Md5-Meq #1 and rMd5-CVI/Md5-Meq #2 using cosmids SN15, P89, B40, SN5-CVI/Md5-Meq, and A6-CVI/Md5-Meq. The rMd5-CVI-Meq infectious virus, in which Md5-Meq was replaced by CVI-Meq, was recovered using the same procedure with cosmids SN5-CVI-Meq plus A6-CVI-Meq.

**Southern blot**. To ensure that the recombination events did not result in major rearrangements in the recombinant viruses’ genomes, DNA from DEF infected with rMd5, rMd5-CVI-Meq, rMd5-CVI-LMeq, rMd5-Md5/CVI-Meq, and rMd5-CVI/Md5-Meq was examined by Southern blot. Briefly, isolated DNA was digested with *EcoR*I, separated on a 1% agarose Tris-borate/EDTA gel, transferred to a nylon membrane, and hybridized with DNA probes, one from total genomic viral DNA (cosmid DNA) and one from a 2.4 kb *Eco*Q fragment labeled with [^32^P]dCTP by the random priming method (DECAprime II kit, Ambion, Austin, TX, USA) [30]. Following hybridization, membranes were washed three times (1–2 h each at 43 °C) with wash buffer [0.2 X saline sodium citrate buffer (SSC) supplemented with 0.1% sodium dodecyl sulfate (SDS)] and then autoradiographed.

**In vitro growth kinetics assay**. Recombinant viruses’ growth kinetics were examined using confluent monolayers of DEF grown in 35 mm dishes. DEF were infected with 100 plaque-forming units (PFU) of each recombinant virus and 2, 3, 4, and 5 days post-infection, cells were trypsinized, diluted 10-fold, seeded onto fresh DEF monolayers, and plaques counted 7 days post-infection (dpi). All titrations were performed twice in duplicate.

**Immunofluorescence assay (IFA)**. To examine the presence of infectious recombinant viruses and Meq expression, infected cells were examined by IFA. Briefly, infected cells were washed with PBS and fixed with ice-cold ethanol–acetone (6:4) solution at room temperature for 10 min. After fixation, cells were air-dried, rehydrated with PBS, and incubated with mouse anti-pp38 monoclonal antibody [31] or rabbit anti-Meq serum [32] at 37 °C for 1 h. Following three washes with PBS, cells were probed with FITC-conjugated goat anti-mouse antibody for anti-pp38-stained cells or FITC conjugated goat anti-rabbit antibody for anti-Meq stained cells for one hour at 37 °C. PBS was used to wash cells 3 times, followed by examination under a fluorescence microscope.

**Immunohistochemistry (IHC)**. To examine the presence of virus replication in lymphoid organs (thymus, spleen, and bursa) and skin (feather follicle epithelium), samples were embedded in optimal cutting temperature compound (Tissue-Tek OCT; Sakura Finetek, Torrance, CA, USA) immediately frozen in liquid nitrogen and kept at −80 °C until use. Eight µm thick sections were prepared from the frozen tissue blocks, fixed with cold acetone at −20 °C for 5 min, and air-dried. The Vectastain ABC kit (Vector Laboratories, Burlingame, CA, USA) was used as per the manufacturer’s instructions. Mouse anti-pp38 monoclonal antibody was used as the detection antibody.

**Generation of single copy (sc) revertant viruses**. Single-copy revertant viruses for each *meq* chimera were generated to study the dominant phenotype of CVI/Md5-Meq and Md5/CVI-Meq vs. Md5 *meq*. Single copy revertant viruses containing parental Md5 *meq* gene were generated using purified DNA from CEF infected with rMd5-CVI/Md5-Meq and rMd5-Md5/CVI-Meq and co-transfected, as indicated before, with parental Md5 *Eco*Q fragment. Individual plaques were collected by trypsinization seven days post-transfection. Cells from individual plaques were split into two aliquots: one was used for DNA extraction and PCR analysis, and the other was used to infect DEF. Mismatch primers (BL1455: 5′- AAA AGG AAT CGT GAC GAC G-3′ and BL1456: 5′- AGT ATC CGA GGG AAA CTT AG-3′), which amplify only the parental Md5 *meq* and not Md5/CVI *meq* or CVI/Md5 *meq* at an annealing temperature of 60 °C, were used to identify revertant viruses. Stocks of single-copy revertant viruses, rMd5-Md5/CVI-MeqSc and rMd5-CVI/Md5-MeqSc, were generated and titrated in DEF and used in in vivo experiments to investigate the dominant phenotype of the Meq domains.

**Chicken experiments.** To evaluate the pathogenicity of recombinant viruses, compared to parental rMd5, 2 different animal experiments were conducted. All animal experiments were conducted according to protocol #2005-53 approved by the Texas A&M University Institutional Animal Care and Use Committee (IACUC).

***Experiment 1*.** Day-old MDV-specific pathogen-free chickens (Charles River SPAFAS, North Franklin, CT, USA) were sorted into experimental groups, wing-banded, and housed in modified Horsfall-Bauer isolators. Four groups of day-old chickens (17 per group) were inoculated subcutaneously with 5000 PFU of rMd5, rMd5-CVI-Meq, rMd5-Md5/CVI-Meq, and rMd5-CVI/Md5-Meq viruses. A group of 8 chickens was inoculated with 5000 PFU of CVI988/Rispens, and a group of 10 chickens was kept uninoculated as a negative control. In addition, three uninoculated chickens were included as contact chickens with each inoculated group (except CVI988/Rispens) to study virus transmission. Chickens were monitored daily for 8 weeks for the development of MD and were necropsied at the time of death or euthanasia to evaluate MD incidence. Mortality before day 12 of age was considered non-specific, and those chickens were not counted in the total for analysis.

Early viral cytolytic infection in spleen, thymus, and bursa samples was examined by IHC in 2 randomly selected chickens from each group at 6 dpi. The reactivation ability of chimeric viruses was examined in the blood of 3 randomly selected chickens from each group at 35 dpi. Buffy coats from heparinized blood were collected and diluted to 10^6^ lymphocytes/mL. A total of 10^6^ lymphocytes were added, in duplicate, to freshly seeded DEF monolayers, and viral plaques were counted 7 days later. To evaluate the replication of chimeric viruses in feather follicle epithelium (FFE), skin from two chickens from rMd5, rMd5-CVI-Meq, rMd5-Md5/CVI-Meq, or rMd5-CVI/Md5-Meq groups was collected at the time of death or the end of the experiment (8 weeks post-inoculation) and examined by IHC for pp38 expression. To investigate the ability of chimeric viruses to transmit among chickens, buffy coats were obtained from contact chickens at 7 weeks, and DEF was co-cultured with 1 × 10^6^ lymphocytes and examined by IFA for pp38 expression 7 days later.

***Experiment 2*.** Nine groups of day-old chickens (15 per group) were inoculated, by the subcutaneous route, with 5000 PFU of the following recombinant viruses: rMd5, rMd5-Md5/CVI-Meq, rMd5-Md5/CVI-Meq #1, rMd5-Md5/CVI-Meq #2, rMd5-CVI/Md5-Meq, rMd5-CVI/Md5-Meq #1, rMd5-CVI/Md5-Meq #2, rMd5-Md5/CVI-Md5-Meq-Sc (single-copy Md5-Meq revertant), and rMd5-CVI/Md5-Md5-Meq-Sc (single-copy Md5-Meq revertant). An uninoculated group of 10 chickens served as a negative control. Chickens were monitored daily for 8 weeks for the development of MD, as well as mortality, and were necropsied at the time of death or euthanasia to evaluate MD incidence. Mortality before day 12 of age was considered non-specific, and those chickens were not counted in the total for analysis.

**Data and Statistical Analysis**. For in vitro growth kinetics, data represent an average of duplicates and were analyzed by ANOVA for each time point. For the virus reactivation assay, blood samples were taken from 3 chickens per group, each tested in duplicate and analyzed by ANOVA. Trends of the chicken survival were examined with LogRank and Wilcoxon tests. All statistical analyses were performed with GraphPad Prism 8 software (GraphPad Software, Inc., La Jolla, CA, USA). A value of *p* < 0.05 was considered statistically significant.

## 4. Discussion

MDV is regarded as one of the most potent oncogenic herpesviruses, as it induces lethal T-cell lymphomas in chickens as early as 2 weeks post-infection, suggesting a direct role of viral proteins in the transformation process [2]. Search for such proteins led to the discovery of Meq, a bZIP protein, which is highly expressed in both tumors and MDV-transformed cell lines [5]. Using an in vitro transformation assay, it was shown that Meq could function as an oncoprotein [6,7,16,17]. Subsequent experiments in chickens with a *meq* knockout virus convincingly proved that Meq is the major oncogenic determinant of MDV [19]. Following these findings, several studies that focused on the mechanisms of Meq-mediated transformation have been reported. Meq has been suggested to transform via the v-Jun pathway [17], and its interaction with CtBP was demonstrated to be critical for transformation [20]. In addition, both homo- and heterodimerization of Meq were shown to contribute to the transformation of lymphocytes [21,22,23]. Distinct amino acid differences observed among Meq proteins of various MDV strains suggest that Meq plays an important role in MDV virulence [33,34,35]. A recent study has demonstrated that amino acid differences among Meq proteins led to enhanced virulence, increased shedding, and ability to overcome vaccinal protection [36].

We previously showed that while Md5-Meq and CVI-Meq proteins can equally transform rat and mouse embryonic fibroblasts, they differ in their transactivation activity of the *meq* promoter [18]. Interestingly, chimeric Meq proteins in which the basic DNA-binding and transcriptional regulatory domains had been swapped between Md5-Meq and CVI-Meq showed intermediate transactivation activity of the *meq* promoter when compared to parental Md5-Meq and CVI-Meq proteins [18]. The main objective of this research was to examine the role of Meq functional domains in MDV transformation. To address this, we constructed and studied the replication, transmission, and transformation properties of recombinant Md5 viruses expressing parental Md5-Meq, CVI-Meq, and chimeric, Md5/CVI-Meq, and CVI/Md5-Meq proteins (Figure 1). In agreement with a previous study showing that Meq is not needed for early cytolytic replication in lymphoid organs [19], our IHC results show that none of the Meq proteins examined altered the early cytolytic replication of recombinant viruses. 

It is well known that MDV is a highly contagious virus and rapidly spreads among susceptible chickens. Several MDV genes, such as U_S_2, U_L_13, gC, and LORF11, have been shown to affect horizontal virus transmission [37,38,39,40,41]. Earlier studies showed that the Meq gene is not involved in the transmission [19]. In addition, depending on the clone used, transmission of CVI988 varies (Isabel Gimeno, personal communication) [42,43]. Our results showed that the Meq recombinant viruses tested were transmitted efficiently, as indicated by virus replication in both FFE (Figure 7) and contact chickens. Therefore, we conclude that CVI-Meq and chimeric Meq proteins do not affect horizontal virus transmission and the poor transmission of CVI988 vaccine strain is not due to amino acid differences in the Meq protein.

The hallmark of MDV infection in chickens is the transformation of T lymphocytes. In this regard, recombinant Md5 viruses expressing CVI-Meq and chimeric Meq proteins induced lymphomas in chickens; however, significant differences were observed in their transformation efficiency, tumor distribution, and tumor size (Figure 4, Figure 5 and Figure 6; Table 2 and Table 3). First, rMd5-Md5/CVI-Meq caused 100% mortality with an average mean death time of 33.3 days, whereas rMd5-CVI/Md5-Meq, on average caused 37% mortality with an average mean death time of 42 days. Second, rMd5-Md5/CVI-Meq induced 100% MD with widely distributed, unusually large tumors in visceral organs (Figure 6), whereas rMd5-CVI/Md5-Meq induced tumor lesions primarily in the nerves and few small tumors in visceral organs, resembling classical MD [44]. On the other hand, although parental rMd5 caused 100% mortality with a mean death time of 37.2 days, tumors were found mainly in the nerves, and visceral tumors were significantly smaller and less frequent. Our study, therefore, shows that rMd5-Md5/CVI-Meq is more pathogenic than rMd5-CVI/Md5-Meq and even parental rMd5, as the tumors were more aggressive, and the mean death time was shorter. More specifically, our data suggest that the basic DNA-binding domain plays a major role in the in vivo transformation ability of Meq, since rMd5-CVI/Md5-Meq caused significantly less MD (37%) than parental rMd5 (100%) and single-copy revertant rMd5-CVI/Md5-Md5-MeqSc (93%). On the other hand, the transcriptional regulatory domain mainly impacts metastasis and the size of visceral tumors, since rMd5-Md5/CVI-Meq caused the same mortality as rMd5 (100%) but a higher incidence of visceral tumors and significantly larger tumor sizes. 

Interestingly, recombinant viruses in which rMd5 *meq* had been replaced with the *meq* gene from CVI988 (rMd5-CVI-Meq) caused MD nerve lesions in 7% of inoculated chickens, whereas in previous studies using an RB1B backbone, expression of CVI-Meq resulted in total abrogation of pathogenesis [26]. We hypothesized that the small differences observed between the two studies might be due to the genetic backbone of the viruses used (Md5 vs. RB1B).

Our results show that the amino acid-rich region (DNA-binding domain) of CVI-Meq, present in CVI/Md5-Meq and CVI-Meq, resulted in predominant lesions in peripheral nerves, as seen in classical MD [44]. On the other hand, expression of the DNA-binding domain of Md5-Meq, present in Md5/CVI-Meq and Md5-Meq, resulted in increased tumors in visceral tumors as seen in acute MD. These results were supported by our studies with single-copy revertant viruses, also suggesting that the Md5-Meq basic amino acid-rich region (DNA-binding domain) has a dominant phenotype driving the virus phenotype toward acute MD, as seen in more recent strains.

Meq is a basic leucine zipper (bZIP) protein that has two major regions: (i) a basic amino acid-rich region that binds to DNA and (ii) a leucine zipper motif, which allows Meq to dimerize. The basic amino acid-rich region allows Meq to bind to specific DNA sequences, called MERE I and MERE II, which are found in various promoters [8,9,10]. The leucine zipper motif of Meq allows it to dimerize with itself, forming homodimers, or with various other bZIP proteins, particularly c-Jun, forming heterodimers. Depending on the dimerization partner and the specific promoter sequences bound, the C-terminal domain of Meq can act as a transactivator or transrepressor of transcription. Both major Meq domains, whether alone or in cooperation, could affect not only the development but also the distribution and size of tumors. First, the basic DNA-binding region might influence which promoters Meq binds to and, in turn, regulate the genes critical for transformation. Md5/CVI-Meq and CVI/Md5-Meq, because of differences in the basic DNA-binding region, could target different promoters, and their DNA-binding affinity could also differ. For example, it is well established that phosphorylation of c-Jun reduces its DNA-binding activity [45,46]. Similarly, it has been shown that the DNA-binding activity of Meq decreased when phosphorylated at serine 42 [12], which is present in both CVI/Md5-Meq and Md5/CVI-Meq. However, CVI/Md5-Meq also has a potential phosphorylation site at position 71 (Figure 1); whether this potential phosphorylation site is responsible for the attenuated phenotype of CVI/Md5-Meq remains to be studied. Second, the function of bZIP proteins is largely affected by their interacting partners, and both homodimerization and heterodimerization of Meq are important for transformation [21,22,23]. Since both Md5/CVI-Meq and CVI/Md5-Meq have identical leucine zipper regions, it is tempting to speculate that this region is less likely responsible for the different phenotypes observed. We have previously shown that Md5/CVI-Meq and CVI/Md5-Meq, unlike CVI-Meq, transactivate the meq promoter, although at a lower level than Md5-Meq [18]. Although Md5/CVI-Meq and CVI/Md5-Meq have comparable transactivation abilities in cell culture, their transactivation ability could not directly correlate with their transformation properties. This is not surprising since a lack of direct correlation between transactivation activity and transformation has also been described for c-Jun [47]. Nevertheless, it is conceivable that the phenotypic differences between Md5-Meq and Md5/CVI-Meq are due to differences in the transcriptional regulatory domain. 

It is important to note that the amino acid sequence of Md5/CVI-Meq, except amino acid 326 (I instead of T) in the transcriptional regulatory domain, is identical to that of RB1B (vvMDV) and GA (vMDV), both of which are known to cause a high incidence of large visceral tumors [48,49]. Since RB1B, GA, and rMd5-Md5/CVI-Meq cause tumors in kidneys and gonads at a higher rate than Md5, this tissue distribution and tumor size could be attributed to their transcriptional regulatory domain. However, it is important to note that the transcriptional regulatory domain alone cannot be responsible for this phenotype because CVI-Meq, which has the same transcriptional regulatory domain as Md5/CVI-Meq but differs in its DNA-binding domain, predominantly causes nerve lesions in the background of the Md5 virus.

In conclusion, our study suggests that the DNA-binding domain of Meq plays an essential role in transformation (tumor incidence), while the transcriptional regulatory domain of Meq influences the distribution and size of MDV-induced tumors. In the future, it is important to generate single-amino acid Meq mutant viruses to understand the role of these amino acids in viral and host gene regulation, leading to T-cell transformation, tumor size, and tissue dissemination. In addition, single-cell RNA sequencing (RNA-Seq) can help determine which genes are up- and down-regulated by the different Meq constructs at the single-cell level.

## Figures and Tables

**Table 1:** Virus reactivation assay from peripheral blood lymphocytes.

Virus	No. of Plaques per One Million Lymphocytes ± SD *
Uninoculated control	0
CVI988/Rispens	11 ± 4 ^c^
rMd5	117 ± 21 ^a^
rMd5-CVI-Meq	6 ± 5 ^c^
rMd5-Md5/CVI-Meq	99 ± 27 ^a^
rMd5-CVI/Md5-Meq	56 ± 7 ^b^

SD: Standard deviation. * Significant differences between groups are marked as letters where different letters represent significant differences at *p* < 0.05.

**Table 2:** Incidence of Marek’s disease in chickens infected with parental and recombinant viruses.

Group	Experiment 1		Experiment 2		Average *	
	No. of Chickens with MD/No. of Total Chickens (%)	Mean Death Time (Days)	No. of Chickens with MD/No. of Total Chickens (%)	Mean Death Time (Days)	No. of Chickens with MD/No. of Total Chickens (%)	Mean Death Time (Days)
Uninoculated Control	0/10 (0)	NA	0/10 (0)	NA	0/20 (0)	NA
CVI988/Rispens	0/8 (0)	NA	ND	NA	0/8 (0)	NA
rMd5	15/15 (100)	38.7	13/13 (100)	35.6	28/28 (100)	37.2
rMd5-CVI Meq	1/15 (7)	55.0	ND	NA	1/15 (7)	55.0
rMd5-Md5/CVI-Meq	15/15 (100)	34.6	14/14 (100)	31.5	56/56 (100)	
rMd5-Md5/CVI-Meq #1	ND	NA	12/12 (100)	35.0		33.3
rMd5-Md5/CVI-Meq #2	ND	NA	15/15 (100)	32.1		
rMd5-Md5/CVI-Md5-MeqSc	ND	NA	13/13 (100)	34.1	13/13 (100)	34.1
rMd5-CVI/Md5-Meq	7/15 (47)	45.0	3/13 (23)	35.6	20/54 (37)	
rMd5-CVI/Md5-Meq #1	ND	NA	4/12 (33)	49.3		42
rMd5-CVI/Md5-Meq #2	ND	NA	6/14 (43)	38.1		
rMd5-CVI/Md5-Md5-MeqSc	ND	NA	14/15 (93)	39.6	14/15 (93)	39.6

ND: not performed; NA: not applicable; * average of both experiments for all clones tested of the same virus.

**Table 3:** Frequency of visceral lesions in chickens inoculated with parental and recombinant viruses.

Virus	Heart (%)	Spleen (%)	Liver (%)	Gonads (%)	Kidney (%)
Uninoculated Control	0/20 (0)	0/20 (0)	0/20 (0)	0/20 (0)	0/20 (0)
CVI988/Rispens	0/8 (0)	0/8 (0)	0/8 (0)	0/8 (0)	0/8 (0)
rMd5-CVI-Meq	0/15 (0)	0/15 (0)	0/15 (0)	0/15 (0)	0/15 (0)
rMd5	7/28 (25)	4/28 (14)	1/28 (4)	1/28 (4)	1/28 (4)
rMd5-Md5/CVI-Meq	24/56 (43)	22/56 (39)	5/56 (9)	9/56 (16)	26/56 (46)
rMd5-Md5/CVI-Md5-MeqSc	3/13 (23)	10/13 (77)	0/13 (0)	7/13 (54)	8/13 (62)
rMd5-CVI/Md5-Meq	1/54 (2)	1/54 (2)	0/54 (0)	0/54 (0)	0/54 (0)
rMd5-CVI/Md5-Md5-MeqSc	3/15 (20)	3/15 (20)	0/15 (0)	0/15 (0)	0/15 (0)

## Data Availability

The original contributions presented in this study are included in the article/Supplementary Material. Further inquiries can be directed to the corresponding author(s).

## Supplementary Materials

The following supporting information can be downloaded at: https://www.mdpi.com/article/10.3390/v17020259/s1, Figure S1: Southern blot analysis of viral genomes.

## References

[1] Zerbini F.M., Siddell S.G., Lefkowitz E.J., Mushegian A.R., Adriaenssens E.M., Alfenas-Zerbini P., Dempsey D.M., Dutilh B.E., Garcia M.L., Hendrickson R.C. (2023). Changes to virus taxonomy and the ICTV Statutes ratified by the International Committee on Taxonomy of Viruses (2023). Arch. Virol..

[2] Calnek B.W. (2001). Pathogenesis of Marek’s disease virus infection. Curr. Top. Microbiol. Immunol..

[3] Witter R.L. (1997). Increased virulence of Marek’s disease virus field isolates. Avian Dis..

[4] Tulman E.R., Afonso C.L., Lu Z., Zsak L., Rock D.L., Kutish G.F. (2000). The genome of a very virulent Marek’s disease virus. J. Virol..

[5] Jones D., Lee L., Liu J.L., Kung H.J., Tillotson J.K. (1992). Marek disease virus encodes a basic-leucine zipper gene resembling the fos/jun oncogenes that is highly expressed in lymphoblastoid tumors. Proc. Natl. Acad. Sci. USA.

[6] Qian Z., Brunovskis P., Rauscher F., Lee L., Kung H.J. (1995). Transactivation activity of Meq, a Marek’s disease herpesvirus bZIP protein persistently expressed in latently infected transformed T cells. J. Virol..

[7] Liu J.L., Lin S.F., Xia L., Brunovskis P., Li D., Davidson I., Lee L.F., Kung H.J. (1999). MEQ and V-IL8: Cellular genes in disguise?. Acta Virol..

[8] Qian Z., Brunovskis P., Lee L., Vogt P.K., Kung H.J. (1996). Novel DNA binding specificities of a putative herpesvirus bZIP oncoprotein. J. Virol..

[9] Levy A.M., Izumiya Y., Brunovskis P., Xia L., Parcells M.S., Reddy S.M., Lee L., Chen H.W., Kung H.J. (2003). Characterization of the chromosomal binding sites and dimerization partners of the viral oncoprotein Meq in Marek’s disease virus-transformed T cells. J. Virol..

[10] Brunovskis P., Qian Z., Li D., Lee L., Kung H.-J., Silva R.F., Cheng H.H., Coussens P.M., Lee L.F., Velicer L.F., Silva R.F., Cheng H.H., Coussens P.M., Lee L.F., Velicer L.F. (1996). Functional analysis of the MDV basic-leucine zipper product, MEQ. The 5th International Symposium on Mare’s Disease.

[11] Liu J.L., Lee L.F., Ye Y., Qian Z., Kung H.J. (1997). Nucleolar and nuclear localization properties of a herpesvirus bZIP oncoprotein, MEQ. J. Virol..

[12] Liu J.L., Ye Y., Qian Z., Qian Y., Templeton D.J., Lee L.F., Kung H.J. (1999). Functional interactions between herpesvirus oncoprotein MEQ and cell cycle regulator CDK2. J. Virol..

[13] Liao Y., Lupiani B., Bajwa K., Khan O.A., Izumiya Y., Reddy S.M. (2020). Role of Marek’s Disease Virus (MDV)-Encoded US3 Serine/Threonine Protein Kinase in Regulating MDV Meq and Cellular CREB Phosphorylation. J. Virol..

[14] Liao Y., Lupiani B., Izumiya Y., Reddy S.M. (2021). Marek’s disease virus Meq oncoprotein interacts with chicken HDAC 1 and 2 and mediates their degradation via proteasome dependent pathway. Sci. Rep..

[15] Xie Q., Anderson A.S., Morgan R.W. (1996). Marek’s disease virus (MDV) ICP4, pp38, and meq genes are involved in the maintenance of transformation of MDCC-MSB1 MDV-transformed lymphoblastoid cells. J. Virol..

[16] Liu J.L., Ye Y., Lee L.F., Kung H.J. (1998). Transforming potential of the herpesvirus oncoprotein MEQ: Morphological transformation, serum-independent growth, and inhibition of apoptosis. J. Virol..

[17] Levy A.M., Gilad O., Xia L., Izumiya Y., Choi J., Tsalenko A., Yakhini Z., Witter R., Lee L., Cardona C.J. (2005). Marek’s disease virus Meq transforms chicken cells via the v-Jun transcriptional cascade: A converging transforming pathway for avian oncoviruses. Proc. Natl. Acad. Sci. USA.

[18] Ajithdoss D.K., Reddy S.M., Suchodolski P.F., Lee L.F., Kung H.J., Lupiani B. (2009). In vitro characterization of the Meq proteins of Marek’s disease virus vaccine strain CVI988. Virus Res..

[19] Lupiani B., Lee L.F., Cui X., Gimeno I., Anderson A., Morgan R.W., Silva R.F., Witter R.L., Kung H.J., Reddy S.M. (2004). Marek’s disease virus-encoded Meq gene is involved in transformation of lymphocytes but is dispensable for replication. Proc. Natl. Acad. Sci. USA.

[20] Brown A.C., Baigent S.J., Smith L.P., Chattoo J.P., Petherbridge L.J., Hawes P., Allday M.J., Nair V. (2006). Interaction of MEQ protein and C-terminal-binding protein is critical for induction of lymphomas by Marek’s disease virus. Proc. Natl. Acad. Sci. USA.

[21] Suchodolski P.F., Izumiya Y., Lupiani B., Ajithdoss D.K., Gilad O., Lee L.F., Kung H.J., Reddy S.M. (2009). Homodimerization of Marek’s disease virus-encoded Meq protein is not sufficient for transformation of lymphocytes in chickens. J. Virol..

[22] Suchodolski P.F., Izumiya Y., Lupiani B., Ajithdoss D.K., Lee L.F., Kung H.J., Reddy S.M. (2010). Both homo and heterodimers of Marek’s disease virus encoded Meq protein contribute to transformation of lymphocytes in chickens. Virology.

[23] Brown A.C., Smith L.P., Kgosana L., Baigent S.J., Nair V., Allday M.J. (2009). Homodimerization of the Meq viral oncoprotein is necessary for induction of T-cell lymphoma by Marek’s disease virus. J. Virol..

[24] Shamblin C.E., Greene N., Arumugaswami V., Dienglewicz R.L., Parcells M.S. (2004). Comparative analysis of Marek’s disease virus (MDV) glycoprotein-, lytic antigen pp38- and transformation antigen Meq-encoding genes: Association of meq mutations with MDVs of high virulence. Vet. Microbiol..

[25] Lupiani B., Reddy S. Protection Properties of mutant rMd5 viruses expressing the Meq protein of CVI988 vaccine strain. Proceedings of the American Association of Avian Pathologists Conference (AVMA/AAAP).

[26] Conradie A.M., Bertzbach L.D., Bhandari N., Parcells M., Kaufer B.B. (2019). A Common Live-Attenuated Avian Herpesvirus Vaccine Expresses a Very Potent Oncogene. mSphere.

[27] Reddy S.M., Lupiani B., Gimeno I.M., Silva R.F., Lee L.F., Witter R.L. (2002). Rescue of a pathogenic Marek’s disease virus with overlapping cosmid DNAs: Use of a pp38 mutant to validate the technology for the study of gene function. Proc. Natl. Acad. Sci. USA.

[28] Cui X., Lee L.F., Reed W.M., Kung H.J., Reddy S.M. (2004). Marek’s disease virus-encoded vIL-8 gene is involved in early cytolytic infection but dispensable for establishment of latency. J. Virol..

[29] Moriuchi H., Moriuchi M., Smith H.A., Straus S.E., Cohen J.I. (1992). Varicella-zoster virus open reading frame 61 protein is functionally homologous to herpes simplex virus type 1 ICP0. J. Virol..

[30] Sambrook J.R.D. (2001). Molecular Cloning: A Loboratory Manual.

[31] Cui Z.Z., Yan D., Lee L.F. (1990). Marek’s disease virus gene clones encoding virus-specific phosphorylated polypeptides and serological characterization of fusion proteins. Virus Genes..

[32] Lee L.F., Liu J.L., Cui X.P., Kung H.J. (2003). Marek’s disease virus latent protein MEQ: Delineation of an epitope in the BR1 domain involved in nuclear localization. Virus Genes..

[33] Padhi A., Parcells M.S. (2016). Positive Selection Drives Rapid Evolution of the meq Oncogene of Marek’s Disease Virus. PLoS ONE.

[34] Trimpert J., Groenke N., Jenckel M., He S., Kunec D., Szpara M.L., Spatz S.J., Osterrieder N., McMahon D.P. (2017). A phylogenomic analysis of Marek’s disease virus reveals independent paths to virulence in Eurasia and North America. Evol. Appl..

[35] Dunn J.R., Black Pyrkosz A., Steep A., Cheng H.H. (2019). Identification of Marek’s disease virus genes associated with virulence of US strains. J. Gen. Virol..

[36] Conradie A.M., Bertzbach L.D., Trimpert J., Patria J.N., Murata S., Parcells M.S., Kaufer B.B. (2020). Distinct polymorphisms in a single herpesvirus gene are capable of enhancing virulence and mediating vaccinal resistance. PLoS Pathog..

[37] Lee L.F., Silva R.F., Cui X., Zhang H., Heidari M., Reddy S.M. (2007). Characterization of LORF11, a unique gene common to the three Marek’s disease virus serotypes. Avian Dis..

[38] Jarosinski K.W., Margulis N.G., Kamil J.P., Spatz S.J., Nair V.K., Osterrieder N. (2007). Horizontal transmission of Marek’s disease virus requires US2, the UL13 protein kinase, and gC. J. Virol..

[39] Blondeau C., Chbab N., Beaumont C., Courvoisier K., Osterrieder N., Vautherot J.F., Denesvre C. (2007). A full UL13 open reading frame in Marek’s disease virus (MDV) is dispensable for tumor formation and feather follicle tropism and cannot restore horizontal virus transmission of rRB-1B in vivo. Vet. Res..

[40] Jarosinski K.W., Osterrieder N. (2010). Further analysis of Marek’s disease virus horizontal transmission confirms that U(L)44 (gC) and U(L)13 protein kinase activity are essential, while U(S)2 is nonessential. J. Virol..

[41] Krieter A., Ponnuraj N., Jarosinski K.W. (2020). Expression of the Conserved Herpesvirus Protein Kinase (CHPK) of Marek’s Disease Alphaherpesvirus in the Skin Reveals a Mechanistic Importance for CHPK during Interindividual Spread in Chickens. J. Virol..

[42] Islam T., Renz K.G., Walkden-Brown S.W., Ralapanawe S. (2013). Viral kinetics, shedding profile, and transmission of serotype 1 Marek’s disease vaccine Rispens/CVI988 in maternal antibody-free chickens. Avian Dis..

[43] Ralapanawe S., Renz K.G., Burgess S.K., Walkden-Brown S.W. (2016). Field studies of the detection, persistence and spread of the Rispens CVI988 vaccine virus and the extent of co-infection with Marek’s disease virus. Aust. Vet. J..

[44] Purchase H.G., Biggs P.M. (1967). Characterization of five isolates of Marek’s disease. Res. Vet. Sci..

[45] Boyle W.J., Smeal T., Defize L.H., Angel P., Woodgett J.R., Karin M., Hunter T. (1991). Activation of protein kinase C decreases phosphorylation of c-Jun at sites that negatively regulate its DNA-binding activity. Cell.

[46] Radler-Pohl A., Gebel S., Sachsenmaier C., Konig H., Kramer M., Oehler T., Streile M., Ponta H., Rapp U., Rahmsdorf H.J. (1993). The activation and activity control of AP-1 (fos/jun). Ann. N. Y. Acad. Sci..

[47] Havarstein L.S., Morgan I.M., Wong W.Y., Vogt P.K. (1992). Mutations in the Jun delta region suggest an inverse correlation between transformation and transcriptional activation. Proc. Natl. Acad. Sci. USA.

[48] Eidson C.S., Schmittle S.C. (1968). Studies on acute Marek’s disease. I. Characteristics of isolate GA in chickens. Avian Dis..

[49] Schat K.A., Calnek B.W., Fabricant J. (1982). Characterisation of two highly oncogenic strains of Marek’s disease virus. Avian Pathol..

