# Supplementary material for: Marek’s Disease Virus (MDV) Meq Oncoprotein Plays Distinct Roles in Tumor Incidence, Distribution, and Size
Source: Viruses. 2025 Feb 14;17(2):259. doi: 10.3390/v17020259 (PMC11860637; doi:10.3390/v17020259)
Supplement: Supplementary file 1 [file viruses-17-00259-s001.zip › viruses-3422716-supplementary.pdf]

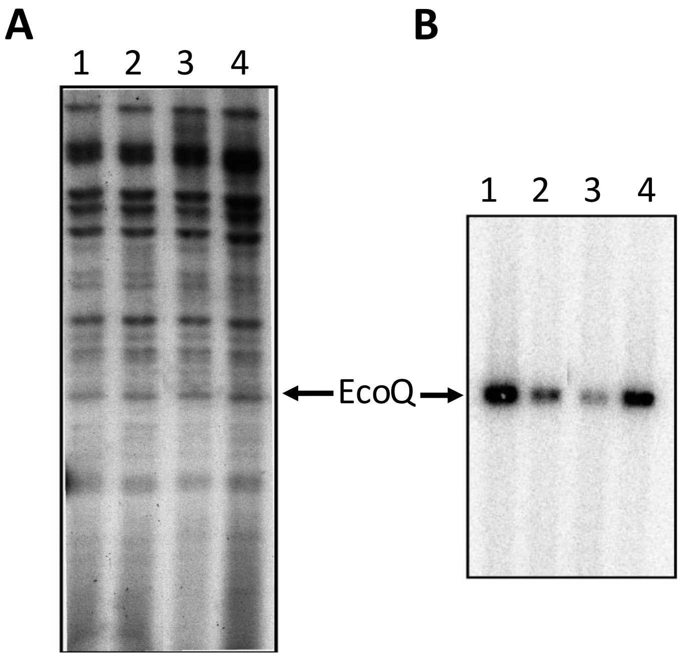

Figure S1. **Southern blot analysis of viral genomes**. DNA isolated from DEF infected with parental and chimeric viruses was digested with *EcoR*I and transferred to nylon membranes. Membranes were probed with either radio-labeled viral genome (**A**) or *Eco*Q fragment (**B**). Lanes: (1) rMd5, (2) rMd5-CVI-Meq, (3) rMd5-Md5/CVI-Meq, and (4) rMd5-CVI/Md5-Meq.
